# Multiplex *FAD2/FATB* Editing Generates Ultra‐High‐Oleic, Low‐Saturate Soybean With Increased Seed Fatty Acid Content

**DOI:** 10.1111/pbi.70758

**Published:** 2026-09-14

**Authors:** Won Nyeong Kim, Yu‐Ri Choi, Hye Jeong Kim, Young‐Soo Chung, Hyun Uk Kim

**Affiliations:** ^1^ Department of Bioindustry and Bioresource Engineering Sejong University Seoul Republic of Korea; ^2^ Department of Molecular Biology Sejong University Seoul South Korea; ^3^ Department of Biomaterials Engineering, College of Natural Resources and Life Sciences Dong‐A University Busan Republic of Korea; ^4^ Institute for Advanced Plant Breeding and Phytochemicals (IAPBP) Sejong University Seoul Republic of Korea

**Keywords:** FAD2, FATB, high‐oleic, lipid turnover, soybean, TAG accumulation

## Abstract

Oleic acid (18:1), saturated fatty acid (SFA), and polyunsaturated fatty acid (PUFA) levels are important traits for storage stability and edibility. In this study, we aimed to develop high‐oleic soybean ( *Glycine max* ) by simultaneously targeting fatty acid desaturase 2 (*FAD2*) and fatty acyl‐ACP thioesterase B (*FATB*) gene families using CRISPR/Cas9. Considering the paleopolyploid genome of soybean, multiple sgRNAs were designed to target *GmFAD2‐1*, *GmFAD2‐2*, and *GmFATB* genes expressed during seed development. Compared with targeting *GmFAD2‐1* alone (~83%), additional editing of *GmFAD2‐2B, GmFAD2‐2C,* and *GmFATB1a* increased the 18:1 fatty acid content to over 90%. Editing of *GmFATB* significantly reduced the SFA content by more than 40% compared with wild‐type (WT). Reduced absolute SFA content was also observed in GmFAD2‐only edited lines with decreased *GmFATB* expression. Notably, all evaluated high‐oleic genotypes in the Williams82‐background showed higher mean total fatty acid (TFA) content than the WT, both per unit seed mass and per seed. Correlation analysis of 805 individual seed profiles from Williams82‐background lines further characterized the relationship between 18:1 fatty acid composition and TFA content per unit seed mass. Gene expression analysis revealed no consistent increase in triacylglycerol (TAG) synthesis genes, whereas the expression of *sugar‐dependent 1* (*SDP1*) was reduced. In contrast, genes involved in phosphatidic acid (PA) metabolism, including *diacylglycerol kinase* (*DGK*) and *phospholipase D* (*PLD*), were partially upregulated. These transcriptional changes support a hypothesis that altered lipid turnover may contribute to the higher TFA phenotype. Collectively, this study defines effective multiplex target combinations for ultra‐high oleic, low‐saturated soybean.

## 1 Introduction

Soybean ( *Glycine max* ) is a crop that provides oil and protein for human consumption and is primarily cultivated in North America, South America, and Asia. Approximately 39% of global soybean production occurs in Brazil (Cattelan and Dall'Agnol 2018). China is the largest importer and consumer of soybeans, and its demand has been rapidly increasing with population growth and economic development (Zhang et al. 2025). In addition, the global soybean market was valued at approximately USD 200 billion by 2023 and grew to USD 212 billion by 2024, showing an average annual growth rate of 4.4%, and is projected to reach USD 388 billion by 2034 (Barboza Martignone et al. 2024). Soybean oil is industrially utilized as a raw material for food, feed, and biodiesel production, and its market is expected to continue to expand (Boerema et al. 2016).

Wild‐type (WT) soybean oil contains approximately 13% palmitic acid (16:0), 4% stearic acid (18:0), 20% oleic acid (18:1), 55% linoleic acid (18:2), and 8% linolenic acid (18:3). Its relatively high polyunsaturated fatty acid (PUFA) content leads to rapid oxidation and, consequently, a short storage period (Martin‐Rubio et al. 2020). Previous studies have shown that higher levels of PUFA increase susceptibility to oxidation due to unsaturated bonds, leading to accelerated degradation during storage. In this process, primary oxidation products, such as peroxides, and secondary oxidation products, such as aldehydes and ketones, are formed (Choe and Min 2006), which may negatively affect the stability and nutritional quality of soybean oil. Accordingly, studies on oil modification in soybeans have primarily focused on reducing polyunsaturated and saturated fatty acid (SFA) contents while increasing 18:1 composition to improve oil stability and nutritional quality. The most common strategy involves editing the soybean fatty acid desaturase 2 (*FAD2*) gene, which catalyses the formation of 18:2 fatty acids. This approach blocks the conversion of 18:1 to 18:2 fatty acids, thereby increasing the 18:1 composition, while significantly reducing PUFA levels. Indeed, the analysis of a high‐oleic mutant generated by X‐ray irradiation revealed a defect in the DNA sequence of *GmFAD2‐1A* (Anai et al. 2008). In that study, targeting a single *GmFAD2‐1A* gene increased the 18:1 composition to approximately 50%. Furthermore, Plenish (DuPont Pioneer) was developed through RNAi‐mediated suppression of *FAD2‐1* and increased 18:1 composition approximately 75% (Kinney 1996). Other studies reported that the transgenic suppression of *FAD2‐1* can increase 18:1 composition approximately 80% or higher (Buhr et al. 2002; Mroczka et al. 2010). Targeted genome editing of these genes has also been reported. Calyno (Calyxt), the TALEN‐mediated mutations of *GmFAD2‐1A* and *GmFAD2‐1B*, showed increased seed 18:1 composition to approximately 80% (Haun et al. 2014), and subsequent stacking with *FAD3* mutations further reduced 18:2 and 18:3 contents while maintaining high 18:1 composition (Demorest et al. 2016). More recently, simultaneous CRISPR/Cas9 editing of *GmFAD2‐1A* and *GmFAD2‐1B* generated double null mutants with more than 80% 18:1 composition (Do et al. 2019). Another strategy to increase the 18:1 composition is to prevent precursor flux at the stages prior to 18:1 fatty acid formation. Fatty acyl carrier protein thioesterase B (*FATB*) converts 16:0‐ACP and 18:0‐ACP into 16:0 and 18:0 free fatty acids (FFA), which are then exported to the cytosol and converted into 16:0‐CoA and 18:0‐CoA, respectively. For example, Vistive Gold (MON 87705), generated by RNAi‐mediated suppression of *FAD2‐1* and *FATB*, contains approximately 71.7% of 18:1% and 6.8% SFA. However, several studies have reported that *FATB* KO generally impairs plant growth and, in soybean, can induce male sterility (MS) (Ma et al. 2021).

Soybean is a paleopolyploid species that underwent genome duplication events approximately 59 million and 13 million years ago, and now exhibits diploid‐like behaviour (Shoemaker et al. 2006). One characteristic of such polyploidy is the presence of multiple homologous genes, which makes it difficult to achieve complete knockout of all target genes, thereby obtaining a clear phenotype. Multiplex editing to secure a clear phenotype introduces additional limitations, as it becomes difficult to independently assess the contribution of each gene to the observed phenotype.

In this study, in addition to the two major seed‐specific genes *GmFAD2‐1A* and *GmFAD2‐1B*, we targeted additional seed‐expressed *GmFAD2‐2* and *GmFATB* genes using multiplex CRISPR/Cas9 editing. Through the development and field propagation of diverse edited lines, we generated an allelic series with seed 18:1 composition ranging from approximately 50% to over 90% and evaluated the contributions of different *GmFAD2* and *GmFATB* combinations to seed fatty acid composition. We further examined the reduction in absolute SFA content observed in *GmFAD2*‐only edited lines in association with *GmFATB* expression. In Williams82‐derived high‐oleic lines, TFA content was evaluated on both a seed‐mass and per‐seed basis, and the relationship between 18:1 composition and TFA content was further characterized using 805 individual seed fatty acid profiles from the *T*_4_ and *T*_5_ generations. Finally, expression analyses of genes associated with TAG synthesis and turnover and phosphatidic acid metabolism were performed to explore potential metabolic factors associated with the higher TFA phenotype. These analyses provide a basis for defining effective multiplex target combinations for ultra‐high oleic, low‐saturated soybean and for proposing a potential link between high‐oleic phenotype and altered lipid turnover during late seed development.

## 2 Results

### 2.1 Identification of Soybean *FAD2* and *FATB* Gene Families and Multiplex CRISPR/Cas9 Editing

**FIGURE 1:**
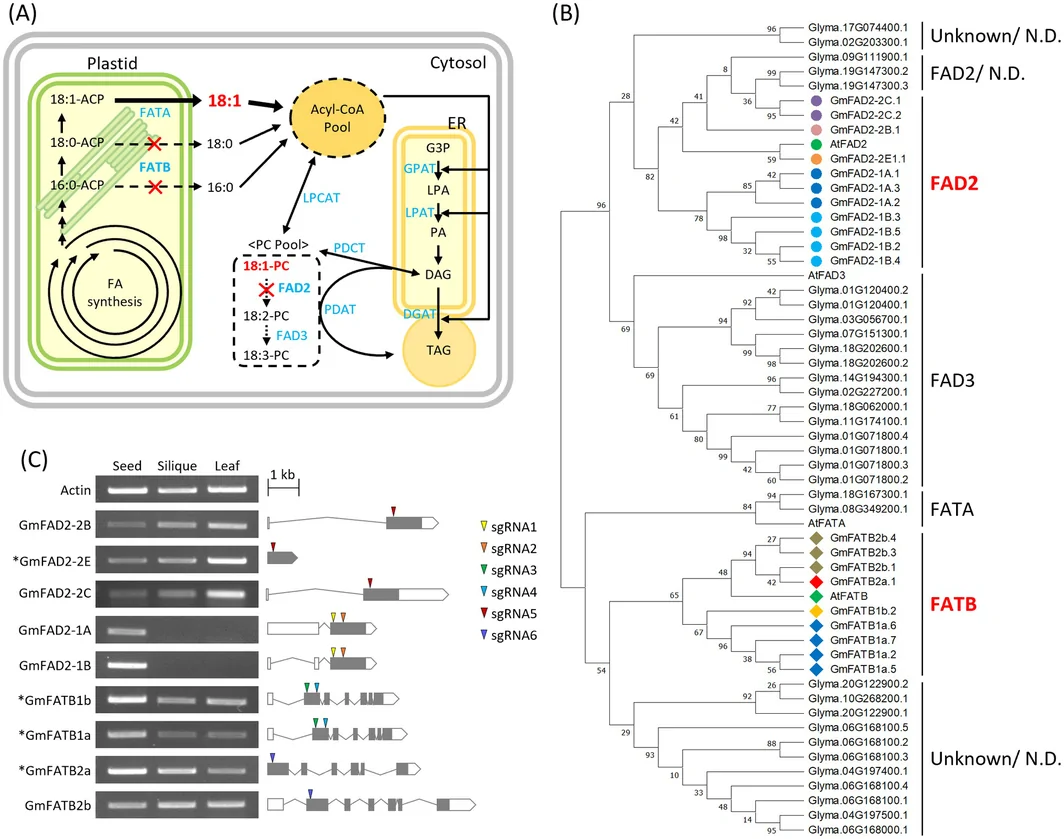
Strategy for high‐oleic, low‐saturated fatty acid biosynthesis and identification of target genes (A) Schematic representation of fatty acid and triacylglycerol biosynthesis and strategy for high‐oleic, low‐saturated fatty acid production. Enzyme names are coloured with cyan. Blocked pathway has indicated with red cross. ACP, Acyl Carrier Protein; CoA, Coenzyme A; DAG, Diacylglycerol; DGAT, Diacylglycerol acyltransferase; ER, Endoplasmic Reticulum; FAD2/3, Fatty Acid Desaturase 2/3; FATA/B, Fatty Acyl‐ACP Thioesterase A/B; G3P, Glycerol‐3‐phosphate; GPAT, Glycerol‐3‐phosphate acyltransferase; LPA, Lysophosphatidic acid; LPAT, Lysophosphatidic acid acyltransferase; LPCAT, Lysophosphatidylcholine acyltransferase; PA, Phosphatidic acid; PC, Phosphatidylcholine; PDAT, Phospholipid: Diacylglycerol acyltransferase; PDCT, Phosphatidylcholine: Diacylglycerol cholinephosphotransferase; TAG, Triacylglycerol. (B) Phylogenetic tree with splicing variant of *Glycine max* FADs and FATs protein sequences with *Arabidopsis thaliana* proteins as control. Target genes including controls were indicated with colour index. Genes showing no detectable expression in seeds, based on the *G. max* RNA‐seq atlas, are denoted as N.D. (Not detected). Gm, *G. max* ; At, *A. thaliana* . The tree was constructed using the maximum‐likelihood method with 1000 bootstrap replicates. (C) Gene expression analysis and gene structure with sgRNA and target location. Genes located on the reverse strand are indicated by an asterisk. The 5′ UTR, 3′ UTR, exon, and intron are indicated with normal white box, white box with pointing tip, grey box, and grey line, respectively.

To increase the 18:1 composition while simultaneously reducing SFA in soybean seeds, *GmFAD2* and *GmFATB* genes involved in fatty acid biosynthesis were targeted for knockout using CRISPR/Cas9 (Figure 1A). Because soybean is a paleopolyploid species, *GmFAD2* and *GmFATB* genes are present as duplicated copies in the genome. To identify these genes in soybean, BLAST analysis was performed using *Arabidopsis thaliana* FAD2 and FATB protein sequences as queries. Phylogenetic analysis was conducted using the identified protein sequences, and *Arabidopsis* FAD3 and FATA protein sequences were used to identify closely related homologous genes. Through this analysis, protein sequences clustered with AtFAD3 and AtFATA, and genes grouped with FAD2 and FATB, but not expressed in seeds, were identified and excluded. As a result, a total of five genes homologous to *AtFAD2* were identified: *GmFAD2‐1A* (Glyma.10G278000), *GmFAD2‐1B* (Glyma.20G111000), *GmFAD2‐2B* (Glyma.19G147400), *GmFAD2‐2C* (Glyma.03G144500), and *GmFAD2‐2E* (Glyma.15G195200). In addition, four genes homologous to *AtFATB* were identified: *GmFATB1a* (Glyma.05G012300), *GmFATB1b* (Glyma.17G120400), *GmFATB2a* (Glyma.04G151600), and *GmFATB2b* (Glyma.06G211300) (Figure 1B). To examine the expression patterns of the identified genes, RT‐PCR was performed using the stem, seed, pod, and leaf tissues. The results showed that *GmFAD2‐1A* and *GmFAD2‐1B* expressions were seed‐specific, whereas *GmFAD2‐2B*, *GmFAD2‐2C*, and *GmFAD2‐2E* were expressed in both seeds and leaves. All four *GmFATB* genes were expressed in both the seeds and leaves (Figure 1C). Based on these results, four genes (*GmFAD2‐1A*, *GmFAD2‐1B*, *GmFATB1a*, and *GmFATB1b*) that appeared to play major roles in the seeds were classified as the major group, whereas the remaining five genes were classified as the minor group. Subsequently, sgRNAs were designed to target as many genes as possible within each group. Two sgRNAs per gene were assigned to the major group, and one sgRNA per gene was assigned to the minor group. As a result, a total of four sgRNAs were allocated to the major group and two sgRNAs to the minor group (Figure 1C).

### 2.2 Construction of Multi‐sgRNA CRISPR/Cas9 Vector and Propagation of Transgenic Plant Through the *T*_0_ _−_ *T*_5_ Generation

**FIGURE 2:**
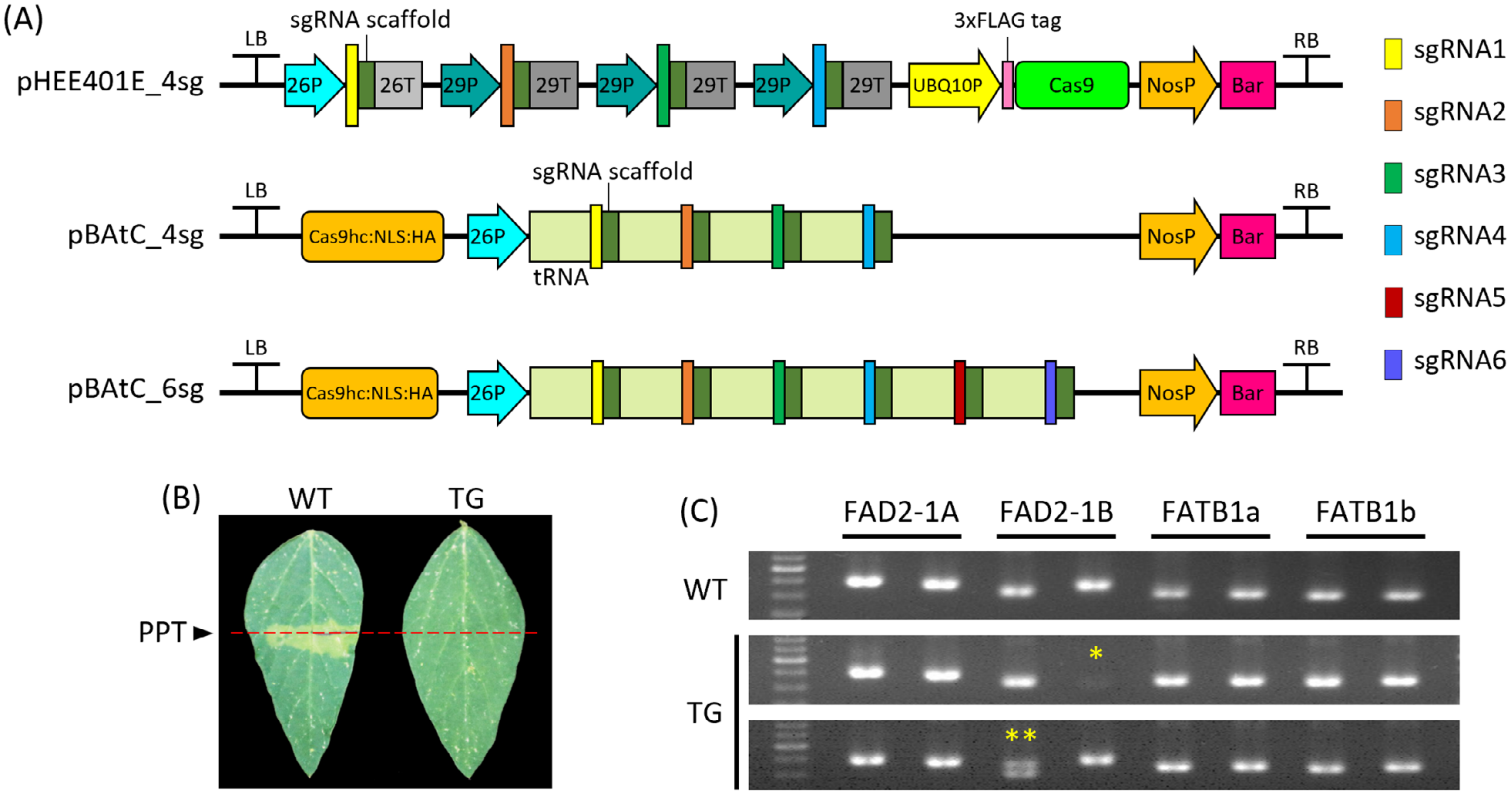
Schematic construct of multi‐sgRNA CRISPR/Cas9 and selection of transgenic line (A) Schematic representations of the T‐DNA regions of the three multi‐sgRNA vectors. sgRNAs were coloured as figure legend. Promoters are indicated with arrow shape. tRNA and sgRNA scaffold sequence for polycistronic sgRNA expression are indicated with pale green and dark green, respectively. LB, Left border; RB, Right border; 26P, U6‐26 promoter; 26T, U6‐26 terminator; 29P, U6‐29 promoter; 29T, U6‐29 terminator; UBQ10P, Ubiquitin‐10 promoter; NosP, Nos promoter; Bar, Bialaphos resistance gene; Cas9hc, Cas9 human‐codon optimized; NLS, Nuclear localization signal; HA, Hemagglutinin tag. (B) *T*_0_ generation transgenic line selection by PPT leaf painting assay. The painted location are indicated with red dashed line. WT, wild‐type (Williams82); TG, Transgenic line. (C) Example of sgRNA target site genotype. Representative products from the second round of nested PCR used for NGS library preparation are shown. The deletion over the detectable window size and segregating heterozygous deletion are indicated with single and double asterisk, respectively.

Previously assigned sgRNA sets were inserted into two vectors. Three constructs were generated: pHEE401E_4sg and pBAtC_4sg, each containing four sgRNAs targeting the major gene group, and pBAtC_6sg, targeting both the major and minor gene groups (Figure 2A). To screen the transgenic plants for these vectors, a PPT leaf painting assay was performed. Chlorosis was observed in the WT plants, whereas the transgenic plants exhibited resistance to PPT, confirming vector transformation (Figure 2B). Genomic DNA (gDNA) was extracted from the leaves of PPT‐positive transgenic plants and library PCR was conducted to evaluate indel efficiency. During library preparation, PCR results revealed large and segregating deletions in the transgenic lines (Figure 2C), indicating that the introduced sgRNAs targeted the intended genomic sites.

Following the selection of *T*_0_ high‐oleic soybean transgenic plants, generational advancement and phenotypic analyses were conducted over 6 years, including 2 years in the greenhouse up to the *T*_2_ generation and 4 years in the LMO field up to the *T*_5_ generation (Figure S1; Tables S1–S11). A total of 16, 26, and 29 *T*_0_ transgenic lines were obtained using the pHEE401E_4sg, pBAtC_4sg, and pBAtC_6sg vectors, respectively. All 71 lines were subjected to indel frequency analysis, and *T*_1_ seeds were collected. Cotyledon samples from *T*_1_ seeds were analysed using gas chromatography (GC) to determine fatty acid composition (Tables S1 and S2). To select lines for advancement, individuals with the highest indel frequencies and 18:1 composition were chosen from each vector group. Specifically, 20, 15, and 15 individuals were selected from the three representative *T*_0_ lines for pHEE401E_4sg, pBAtC_4sg, and pBAtC_6sg, respectively, and advanced to the *T*_1_ generation (Table S3). Indel analysis was conducted on 50 *T*_1_ lines, and *T*_2_ seeds were harvested from each line for GC analysis (Tables S3 and S4). Based on these results, 65 individuals from seven pHEE401E_4sg lines, 62 individuals from five pBAtC_4sg lines, and 31 individuals from five pBAtC_6sg lines, all showing high indel frequencies and 18:1 composition, were advanced to the next generation. A total of 158 *T*_2_ individuals were subjected to indel analysis (Table S5), and 1149 *T*_3_ seeds, including transgenic and WT seeds as controls, were analysed using GC (Table S6). The *T*_3_ generation consisted of 80 individuals from eight pHEE401E_4sg lines, 70 individuals from seven pBAtC_4sg lines, and 60 individuals from six pBAtC_6sg lines (Table S7). In addition, *T*_2_ seeds were sown in the same year, advancing 21 pHEE401E_4sg, 8 pBAtC_4sg, and 6 pBAtC_6sg individuals, resulting in 245 individuals across the *T*_2_ and *T*_3_ generations. From these, *T*_3_ and *T*_4_ seeds were harvested, and 388 seeds were selected for quantitative GC analysis (Table S8). To obtain diverse indel combinations, the observed indel patterns were categorized into 22 types, and *T*_4_ seeds with high 18:1 composition was selected for each type. The *T*_4_ generation included 64 individuals from five pHEE401E_4sg lines, 28 individuals from three pBAtC_4sg lines, and 72 individuals from five pBAtC_6sg lines (Table S9). Additionally, 28 individuals from the two pHEE401E_4sg lines in the Maverick‐background of the *T*_3_ generation advanced (Table S9). *T*_5_ seeds were harvested from these individuals, and 619 seeds were analysed using GC (Table S10). For further advancement, emphasis was placed on the pBAtC_6sg construct, which allowed for diverse indel combinations. Consequently, 150 individuals from five fixed pHEE401E_4sg lines, 30 individuals from a fixed pBAtC_4sg line, and 129 individuals from seven segregating pBAtC_6sg lines were identified (Table S11).

### 2.3 Fatty Acid Composition and Content of *GmFAD2* and *GmFATB* Editing Types

Transgenic lines were advanced to the *T*_5_ generation to obtain a broader spectrum of edited genotypes (Figure S1). Transgenic plants were screened by deep sequencing of leaf tissues from the *T*_*n*_ generation, followed by GC analysis of seeds obtained from the corresponding *T*_*n*+1_ generation. Finally, 24 independent lines representing 15 distinct indel types were identified (Table 1). These lines were classified based on a combination of edits in the *GmFAD2‐1*, *GmFAD2‐2*, and *GmFATB1* gene families. No individuals were obtained in which all nine target genes were simultaneously edited, and *GmFATB2* was not edited in any of the lines.

**TABLE 1:** List of homozygous GmFAD2/FATB mutants and indel types.

K.O. type (FAD2‐1/FAD2‐2/FATB1)	Gene (Indel type)							Cultivar
	GmFAD2‐1A	GmFAD2‐1B	GmFAD2‐2C	GmFAD2‐2E	GmFAD2‐2B	GmFATB1a	GmFATB1b	
2‐1a/‐/‐	0+14d	—	—	—	—	—	—	W
2‐1ab/‐/‐	4d+0	1i+0	—	—	—	—	—	
	1i+0	8d+0	—	—	—	—	—	
2‐1a'b/‐/‐	6d+0	1i+0	—	—	—	—	—	
2‐1ab'/‐/‐	1i+0	6d+0	—	—	—	—	—	
2‐1a'b'/‐/‐	3d+0	6d+0	—	—	—	—	—	
2‐1ab/2‐2c/‐	0+8d	5d+7d	1i	—	—	—	—	
	0+8d	1i+7d	1i	—	—	—	—	
	0+8d	21i	4d	—	—	—	—	
2‐1ab/2‐2ce/‐	LD+5d	0+2d	5d	4d	—	—	—	
	4d+5d	5d+7d	1i	2d	—	—	—	
2‐1ab/2‐2bc/‐	0+8d	5d+7d	4d	—	5d	—	—	
	4d+5d	7d	1i	—	5d	—	—	
*2‐1ab/‐/1a*	5d+11d	LD	—	—	—	1i+0	—	M
*2‐1ab/‐/1b*	0+31d	6d+3d	—	—	—	—	0+1d	W
	LD	RI	—	—	—	—	351i+15d	
	5d+49d	4d+31d	—	—	—	—	7d+0	M
*2‐1ab/‐/1ab*	5d+49d	4d+31d	—	—	—	4d+0	7d+0	
	5d+49d	4d+10d	—	—	—	5d+0	7d+0	
*2‐1ab/‐/1a'b*	5d+49d	4d+31d	—	—	—	3d+0	7d+0	
	4d+10d	43i+8d	—	—	—	6d+0	6d+1d	W
*2‐1ab/2‐2c/1a*	4d+8d	5d+7d	1i	—	—	1d+0	—	
*2‐1ab/2‐2ce/1a*	4d+5d	5d+7d	1i	2d	—	1d+0	—	
*2‐1ab/2‐2bc/1a*	4d+8d	5d+7d	1i	—	1i	1d+0	—	

*Note:* In the K.O. type column, the name of the type is a combination of the knockout status of FAD2‐1, FAD2‐2, and FATB1, separated with a slash as follows: Lowercase letter, knockout, lowercase letter with an apostrophe, in‐frame knockout. Single “—” indicates wild‐type. In the gene column, target genes with two editing sites are indicated as (1st site indel) + (2nd site indel). The indel type number indicates the edited number of base pairs (e.g., 1i+7d equals a 1 bp insertion at the 1st site and a 7 bp deletion at the 2nd site).

Abbreviations: d, deletion; i, insertion; LD, large deletion; M, Maverick; RI, reverse insertion; W, Williams82.

The indel patterns of the multiplex gene‐edited lines were further analysed (Table S12). First, a wide range of indel events were observed in lines with edits (from 1 bp insertions to deletions of up to 14 bp) in *GmFAD2‐1A* and *GmFAD2‐1B*. Notably, in‐frame deletion variants of 3 and 6 bp, which may retain protein function while altering activity, were obtained. Second, lines simultaneously targeting *GmFAD2‐1A*, *B* and *GmFAD2‐2C, E*, *B* exhibited complex editing patterns, including large and multiple deletions, such as 5 and 7 bp. Multiplex mutant lines with up to four simultaneously edited *GmFAD2* genes (*GmFAD2‐1A*, *B*, *GmFAD2‐2C*, *E*, and *GmFAD2‐2C*, *B*) were identified. Third, a group targeting both *GmFAD2‐1* and *GmFATB1* gene families showed strong genomic alterations, including large insertions of up to 351 bp and extensive deletions. Finally, a group of multiplex mutants in which five genes were simultaneously edited was identified. These lines encompassed edits across *GmFAD2‐1*, *GmFAD2‐2*, and *GmFATB1* gene families, displaying combined mutation patterns ranging from 1 bp insertions to 8 bp deletions at each target locus (Table 1; Table S12).

For notation of indel types, *GmFAD2‐1A* and *GmFAD2‐1B* were abbreviated as *2‐1ab*, *GmFAD2‐2B*, *GmFAD2‐2C*, and *GmFAD2‐2E* as *2‐2BCE*, and *GmFATB1a* and *GmFATB1b* as *1ab*. The types with the highest number of edited genes were *2‐1ab/2‐2ce/1a* and *2‐1ab/2‐2bc/1a*, in which five genes were edited. In‐frame deletions are denoted by adding a prime symbol (′) to the gene abbreviations, such as *2‐1a*′*b*′, *2‐1ab*′, *2‐1a*′*b*, and *2‐1ab/‐/1a*′*b* (Table 1; Table S12).

**FIGURE 3:**
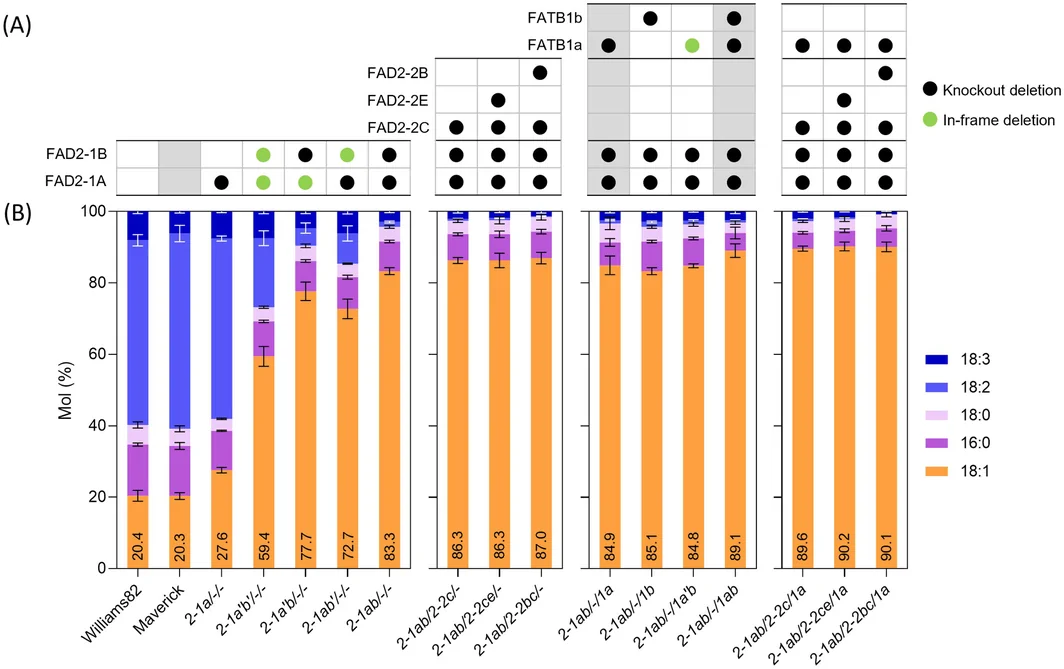
Seed fatty acid profile of wild‐type (WT) and *GmFAD2/GmFATB* mutants (A) Table map for *GmFAD2‐1*, *GmFAD2‐2*, *GmFATB1* gene KO state of each genotype. Knockout and in‐frame deletion are marked with black and green dot, respectively. Williams82‐background line and Maverick‐background line are indicated with white and grey shade, respectively. (B) Seed fatty acid profile of each genotype. Mean value of Oleic acid ratio is indicated on bottom of the bar. *n* > 3. Error bars represent standard deviation (SD).

To evaluate the seed fatty acid profiles across the 15 indel types, GC analysis was performed (Figure 3, Figure S2). The results showed that 18:1 composition varied depending on the number of edited genes in the *GmFAD2* and *GmFATB* families (Figure 3A,B). In lines targeting only *GmFAD2‐1A* and *GmFAD2‐1B*, 18:1 composition increased differentially depending on the *GmFAD2‐1* allele (Figure 3B). The WT 18:1 composition was 20.4% and 20.3% in Williams82 and Maverick, respectively, the *2‐1a′b′/‐/‐* line with in‐frame deletions was 59.4%, and the *2‐1a′b/‐/‐* and *2‐1ab′/‐/‐* lines were 77.7% and 72.7%, respectively. In contrast, the complete knockout line *2‐1ab/‐/‐* exhibited a further increase of 83.3% (Figure 3B).

Additional editing of the *GmFATB1* gene family resulted in changes in the total SFA ratio (Figure S2C). In the *2‐1ab/‐/1a* line, 18:1 was 84.9%, 16:0 was 6.4%, and 18:0 was 6.2%, whereas in the *2‐1ab/‐/1b* line, 18:1 was 85.1%, 16:0 was 8.3%, and 18:0 was 4.1%. In the *2‐1ab/‐/1a′b* line containing an in‐frame knockout, 18:1 was 84.8%, 16:0 was 7.7%, and 18:0 was 3.9%. In the *2‐1ab/‐/1ab* line, in which both *GmFATB1a* and *GmFATB1b* were edited, 18:1 increased to 89.1%, and 16:0 and 18:0 decreased to 4.9% and 2.9%, respectively.

When the *GmFAD2‐2* gene family was additionally edited in the *GmFAD2‐1* background, a further increase in 18:1 composition and a reduction in PUFA were observed (Figure S2D). The *2‐1ab/2‐2c/‐* and *2‐1ab/2‐2ce/‐* lines showed an 18:1 composition of 86.3%, while the *2‐1ab/2‐2bc/‐* line showed 87.0%. In the *2‐1ab/2‐2bc/‐* line, the 18:2 and 18:3 contents were 0.3% and 1.3%, respectively, whereas those in the WT were 51.7% and 8.1%, respectively.

In lines in which the *GmFAD2‐1*, *GmFAD2‐2*, and *GmFATB1* gene families were edited, further increases in 18:1 and decreases in 16:0 were observed (Figure 3; Figure S2C). The *2‐1ab/2‐2c/1a* line showed 18:1 at 89.6%, 16:0 at 4.5%, and 18:0 at 3.2%. The *2‐1ab/2‐2ce/1a* line exhibited the highest 18:1 composition (90.2%), with 16:0 and 18:0 contents of 4.4% and 3.2%, respectively. The *2‐1ab/2‐2bc/1a* line showed 18:1 at 90.1%, 16:0 at 5.2%, and 18:0 at 3.8%. In these multiplex‐edited lines, 18:2 and 18:3 contents remained in the range of 0.1% to 0.8%.

Collectively, additional editing of *GmFAD2‐2B, C* and *GmFATB1a*, along with seed‐specific *GmFAD2‐1A* and *GmFAD2‐1B*, increased 18:1 composition by over 90% (Figure 3). These results indicate that *GmFAD2‐1A* and *GmFAD2‐1B*, as well as *GmFAD2‐2B, C* and *GmFATB*, contribute to the increase in 18:1 composition.

### 2.4 Decreased Seed SFA Accumulation and *GmFATB* Expression in *GmFAD2*‐Exclusively Edited High‐Oleic Lines

**FIGURE 4:**
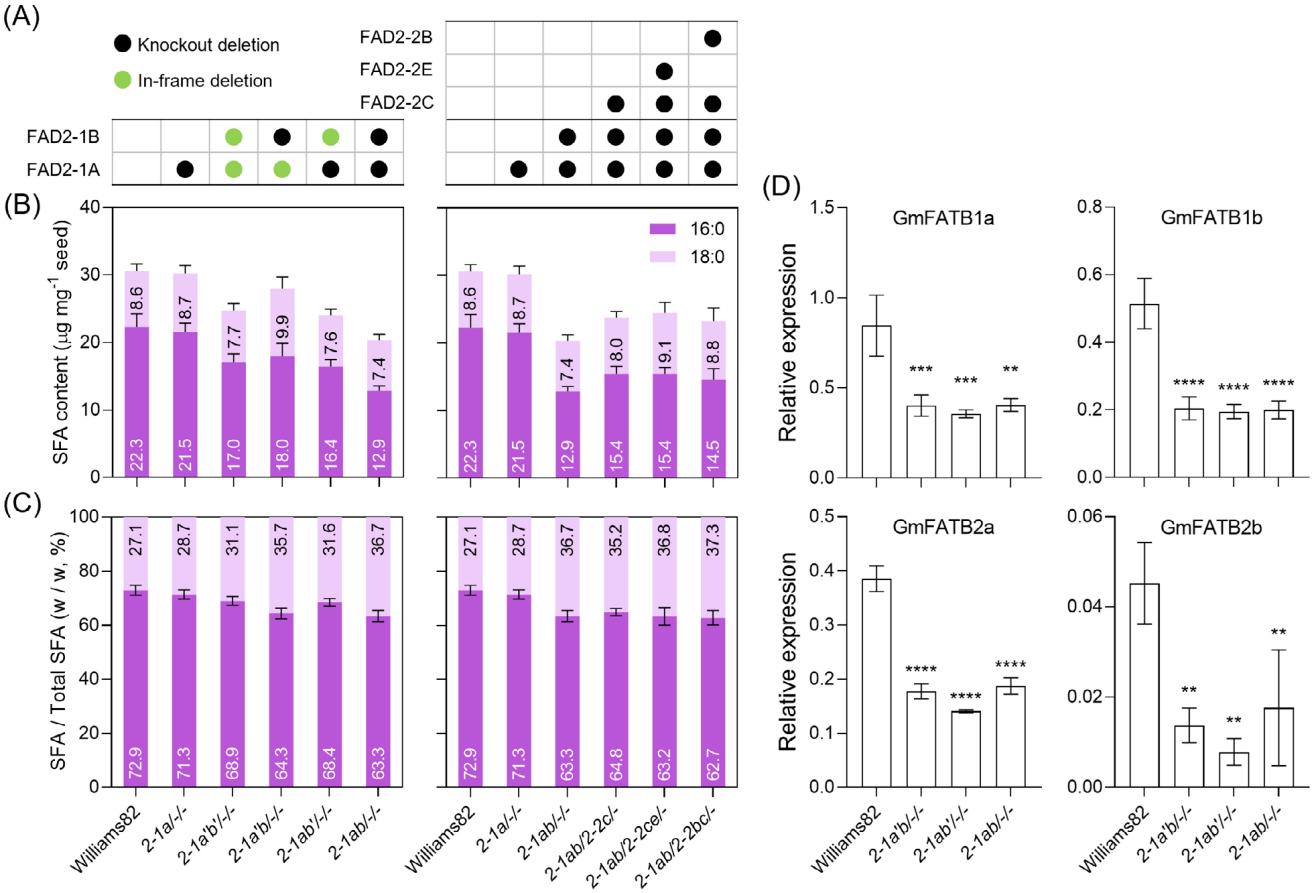
Saturated fatty acid (SFA) profile and *GmFATB* expression (A) Table map for *GmFAD2‐1*, and *GmFAD2‐2* gene KO state of each genotype. Knockout and in‐frame deletion are marked with black and green dot, respectively. Williams82 background line has indicated with white shade. (B) Amount of Stearic and Palmitic acid (SFA content, μg mg^−1^ seed) from *FAD2* gene K.O. types. (C) Percentage of each saturated fatty acid (SFA) by total saturated fatty acid (Total SFA) (D) Relative expression analysis of FATBs. Average of Cons6 and Cons7 gene expression of WT has used as relative standard. *n* = 3. Error bars represent standard deviation (SD). Statistical significance was assessed using one‐way ANOVA followed by Dunnett's multiple‐comparison test for relative gene expression (**p < 0.01, ***p < 0.001, ****p < 0.0001) against the WT.

In lines targeting only the *GmFAD2* gene family, the composition of SFA decreased by approximately 30%–40% compared to that in the Williams82 (WT), reaching 12.3% in the *2‐1ab/‐/‐* line and 11.4% in the *2‐1ab/2‐2bc/‐* line (Figure S2C). Quantitative GC analysis was performed to determine whether this reduction was due to a relative decrease in accumulation caused by increased 18:1 composition. The results showed that WT contained 22.3 μg mg^−1^ seed of 16:0 and 8.6 μg mg^−1^ seed of 18:0, whereas the *2‐1ab/‐/‐* and *2‐1ab/2‐2bc/‐* lines exhibited reduced levels of 12.9 μg mg^−1^ seed and 7.4 μg mg^−1^ seed, and 14.5 μg mg^−1^ seed and 8.0 μg mg^−1^ seed, respectively (Figure 4A,B).

Additionally, to examine the relative contributions of 16:0 and 18:0 to the total SFA, their proportions were calculated against the total SFA content. In the WT, 16:0 and 18:0 accounted for 72.9% and 27.1% of the total SFA, respectively. However, with *GmFAD2* gene editing, the proportion of 16:0 gradually decreased. In the *2‐1ab/‐/‐ line*, the proportion of 16:0 decreased to 63.3%, and further decreased to 62.7% in the *2‐1ab/2‐2bc/‐* line, in which *GmFAD2‐2* genes were additionally edited. In contrast, the proportion of 18:0 increased from 27.1% in the WT to 37.3% in the *2‐1ab/2‐2bc/‐* line (Figure 4C). These results indicate that *GmFAD2* gene editing alone reduced SFA accumulation, with a more pronounced decrease in 16:0 than in 18:0.

To investigate whether SFA reduction was associated with changes in *GmFATB* expression, the expression of *GmFATB* genes was analysed by RT‐qPCR in the seeds of GmFAD2‐1 edited lines, including those with in‐frame knockouts (Figure 4D). The results showed that in all lines with edited *GmFAD2‐1A*,*B* genes, including in‐frame deletions, the expression levels of *GmFATB1a*, *GmFATB1b*, *GmFATB2a*, and *GmFATB2b* were reduced by approximately 40%–70% compared to WT.

These findings showed that reduced *GmFATB* expression was associated with lower SFA accumulation in *GmFAD2*‐edited lines, with a stronger reduction in 16:0 than in 18:0.

### 2.5 Increase of Total Fatty Acid Content in Williams82‐Background High‐Oleic Lines

**FIGURE 5:**
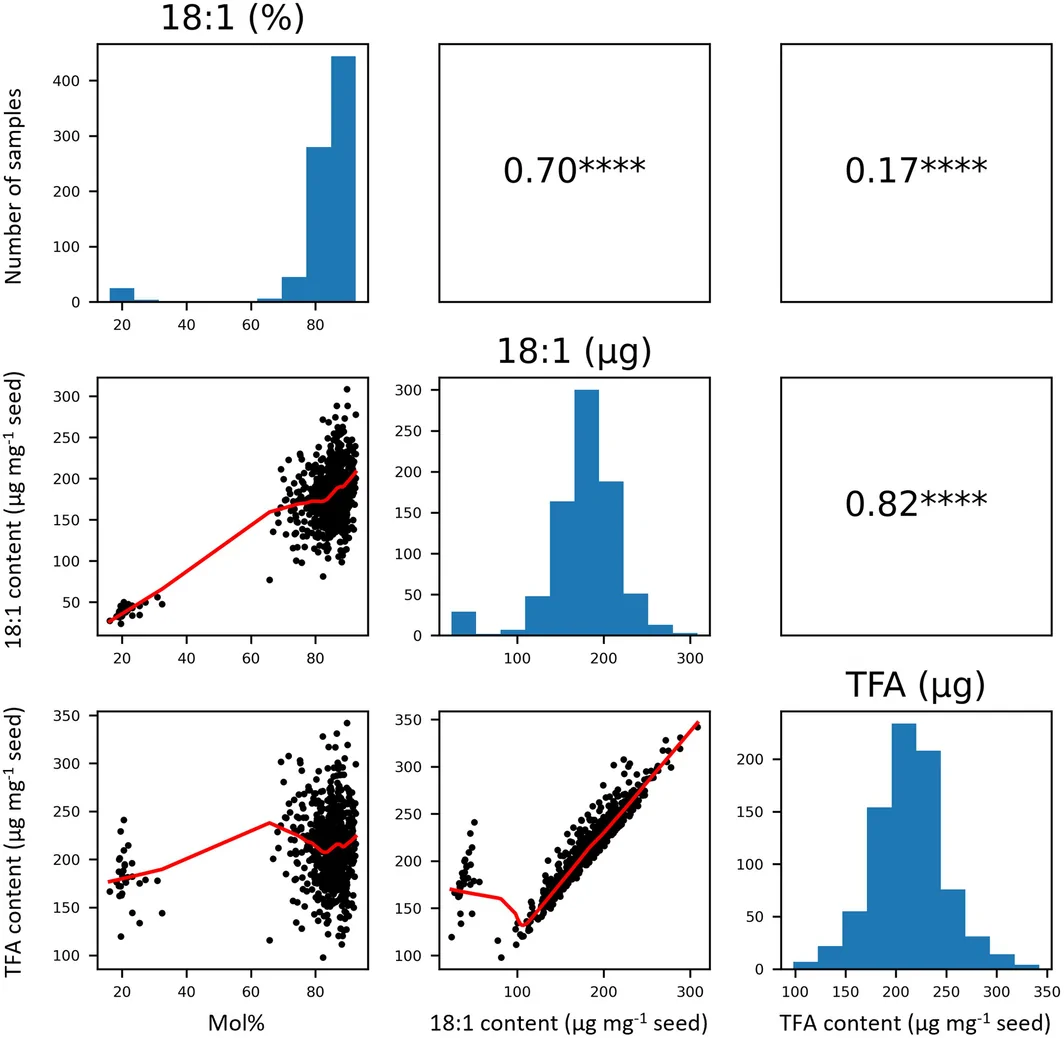
The correlation matrix of oleic acid and total fatty acid among Williams82‐background high‐oleic lines generated by *GmFAD2*/*GmFATB* gene editing The correlation matrix plot of oleic acid ratio, oleic acid amount, and total fatty acid amount. Pearson correlation coefficient (*r*) with asterisk *p*‐value (upper triangle), scatter plot with Locally Weighted Scatterplot Smoothing (LOWESS) regression line (lower triangle), and the distribution histogram of each variable (diagonal line, *Y* axis: Number of data, *X* axis: Unit of each variable) have indicated. Ratio (Mol %) and amount (FA content, μg mg^−1^ seed) of 18:1 and TFA have indicated with each variable name by “(%)” and “(μg)”. Two‐tailed *p*‐value of Pearson correlation coefficient has indicated with asterisk. (****p < 0.0001). TFA, total fatty acid.

To investigate the relationship between fatty acid composition (Mol%) and content (μg mg^−1^ seed) in the high‐oleic lines, Pearson correlation analysis and locally weighted scatterplot smoothing (LOWESS) regression were performed using Williams82‐background line GC data from 805 individual seed samples, including the Williams82 WT, analysed across the *T*_4_ and *T*_5_ generations (Figure 5; Figure S3). The analysis revealed that 18:1 composition and content showed negative correlations with its biosynthetic precursor fatty acids, 16:0 and 18:0 composition, as well as with downstream unsaturated fatty acids, 18:2 and 18:3 composition, with correlation coefficients ranging from *r* = −0.4 to −0.95. Among these, 18:2 composition exhibited the strongest negative correlation with 18:1 composition (*r* = −0.95) (Figure S3). In contrast, metabolically adjacent fatty acids were positively correlated with 16:0, 18:0 composition (*r* = 0.73), and 18:2, 18:3 composition (*r* = 0.81), reflecting their metabolic connectivity. In the analysis based on absolute content per seed unit weight (μg mg^−1^ seed), 18:1 content showed negative correlations with 16:0, 18:3, and 18:2 contents, consistent with the proportional analysis, although the correlations were generally weaker (*r* = −0.09 to −0.65) (Figure S3). However, 18:0 content showed a weak positive correlation with 18:1 content (*r* = 0.11), in contrast to the negative relationship observed in the proportional analysis (Figure S3). When comparing proportion and content within the same fatty acid, strong positive correlations were observed (*r* = 0.7–0.99), with 18:1 showing the weakest (*r* = 0.7) (Figure S3). Total fatty acid (TFA) content (μg mg^−1^ seed) showed a positive correlation with 18:1 composition (*r* = 0.17) and the strongest positive correlation with 18:1 content (*r* = 0.82) (Figure 5).

Additionally, to address the potential confounding effects in the correlation analysis, TFA content was compared according to transformant origin, genetic background, and genotype (Figure S4). In the Williams82‐background, TFA content was consistently higher than that of wild‐type Williams82 across both transformant origins and genotype‐based comparisons (Figure S4A,C). In contrast, Maverick‐background lines showed inconsistent TFA content across generations, and genotype‐based comparisons showed lower TFA content than wild‐type Maverick (Figure S4B,C).

These results indicate that, compared with WT seeds, Williams82‐background high‐oleic lines generated by *GmFAD2* or *GmFAD2*/*GmFATB* editing accumulated higher TFA content.

### 2.6 Increase of Seed TFA in High‐Oleic Line and Analysis of Triacylglycerol Related Genes

**FIGURE 6:**
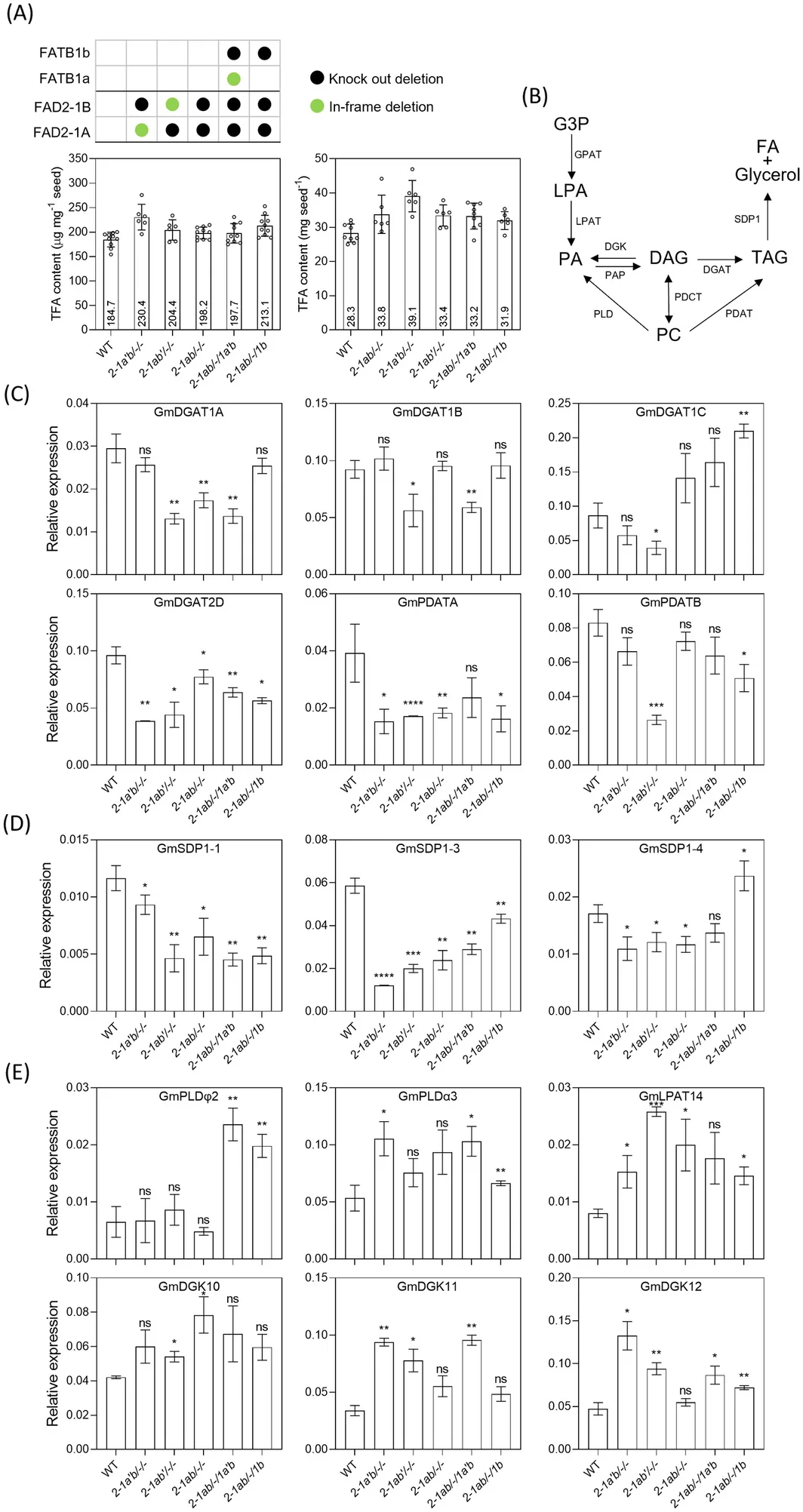
Seed total fatty acid, schematic lipid metabolism pathway and related gene expression (A) TFA content per seed unit weight (μg mg^−1^ seed) and total seed (mg seed^−1^) of five high‐oleic lines with table map for *GmFAD2‐1*, *GmFATB1* gene KO state of each genotype. Knockout and in‐frame deletion are marked with black and green dot, respectively. (B) Schematic pathway of lipid metabolism including synthesis and degradation of TAG. (C) Relative expression analysis of TAG assembly related genes. (D) Relative expression analysis of lipid turnover related genes. (E) Relative expression analysis of PA metabolism related genes. Average of Cons6 and Cons7 gene expression of WT has used as relative standard. *n* = 3. Error bars represent standard deviation (SD). Statistical significance was assessed using one‐way ANOVA followed by Dunnett's multiple‐comparison test for total fatty acid content and relative gene expression (*p < 0.05, **p < 0.01, ***p < 0.001, ****p < 0.0001) against the WT. ns: Not significant. DAG, Diacylglycerol; DGAT, Diacylglycerol acyltransferase; DGK, Diacylglycerol kinase; FA, Fatty acid; G3P, Glycerol‐3‐phosphate; GPAT, Glycerol‐3‐phosphate acyltransferase; LPA, Lysophosphatidic acid; LPAT, Lysophosphatidic acid acyltransferase; PA, Phosphatidic acid; PAP, Phosphatidic acid phosphatase; PC, Phosphatidylcholine; PDAT, Phospholipid: Diacylglycerol acyltransferase; PDCT, Phosphatidylcholine: Diacylglycerol cholinephosphotransferase; PLD, Phospholipase D; SDP1, Sugar‐dependent triacylglycerol lipase 1; TAG, Triacylglycerol.

In the Williams82‐background high‐oleic lines, the TFA content increased by approximately 7%–24% compared with the WT (Figure 6A) on both per unit seed mass (μg mg^−1^ seed) and per seed (mg seed^−1^). Plant and seed phenotypes were evaluated to determine whether this increase was associated with improvements in agronomic traits. Plant height, number of nodes and pods, and seed yield per plant in the LMO field did not differ significantly between the high‐oleic lines and the WT. In the case of branch number, only the *2‐1a′b/‐/‐* line exhibited a higher value than the WT. For seed phenotypes, the *2‐1ab′/‐/‐*, *2‐1a′b/‐/‐*, and *2‐1ab/‐/‐* lines, in which *GmFATB1a* was not edited, showed increased seed size and 100‐seed weight. However, no significant differences in seed number per plant were observed (Figure S5). These inconsistent results indicate that the increase in TFA content was not due to improvements in agronomic traits.

To further explain the increased TFA content, RT‐qPCR analysis was performed on five high‐oleic lines and wild‐type Williams82 (WT) for the genes involved in triacylglycerol (TAG) accumulation (Figure 6B). Analysis of soybean *diacylglycerol acyltransferase* (*GmDGAT*) and *phospholipid: diacylglycerol acyltransferase* (*GmPDAT*), which are directly involved in TAG synthesis, showed that the expression levels of *GmDGAT1A*, *GmDGAT2D*, and *GmPDATA* were reduced to 10%–50% of the WT levels, and *GmPDATB* expression decreased by up to approximately 74% in some lines. In contrast, *GmDGAT1B* and *GmDGAT1C* showed increased expression in only a subset of the lines (Figure 6C; Figure S6).

In contrast, sugar‐dependent 1 (*SDP1*), which is involved in lipid turnover during late seed development (Kelly et al. 2011; Qin et al. 2023), showed 20%–80% reduced expression, except in the *2‐1ab/‐/1b* line (Figure 6D). In soybean, RNAi‐mediated suppression of *SDP1* expression increases TAG content (Kanai et al. 2019). Furthermore, based on previous findings in *A. thaliana* that PHR1‐like7 (*AtPHL7*) regulates *AtSDP1* expression in a phosphatidic acid (PA) dependent manner during seed development (Tang et al. 2026), the expression patterns of PA related genes were analysed. These included Kennedy pathway genes that synthesize de novo PA, glycerol‐3‐phosphate acyltransferase (*GPAT*), and lysophosphatidic acid acyltransferase (*LPAT*), as well as lipid editing PA synthesis genes, phospholipase D (*PLD*), and diacylglycerol kinase (*DGK*). *GmGPAT18*, *22*, *27* (Li et al. 2025) and *GmLPAT4*, *7*, *14* (Wang et al. 2024), *GmPLDα1*, *α2*, *α3*, *φ1*, *φ2* (He et al. 2025), and 10 *GmDGK* genes, which are expressed in the seeds, were analysed (Figure S6; Figure 6E).

Results showed no consistent or significant trends in *GmGPAT* expression. However, *GmLPAT14*, which catalyses PA synthesis from lysophosphatidic acid (LPA), was upregulated (Figure 6E), and *GmLPAT4* and *GmLPAT7*, which were not detected in the WT, were detected in the high‐oleic lines (Figure S6). Additionally, the expression of *GmPLDα3* and *GmPLDφ2*, which are involved in PA production via phosphatidylcholine (PC) degradation, was increased (Figure 6E). Notably, multiple *GmDGK* genes (8, 9, 10, 11, and 12) were consistently upregulated (Figure 6E, Figure S6B).

These results suggest that the higher TFA phenotype was associated with reduced *GmSDP1* expression rather than increased expression of TAG synthesis genes. The altered expression of several PA metabolism genes further suggests a potential association between PA metabolism and the observed transcriptional changes.

## 3 Discussion

FAD2 has been extensively studied in a wide range of plant species, including *A. thaliana* and various oilseed crops. Studies in *Arabidopsis* have shown that *fad2* mutants exhibit greater cold sensitivity than WT plants, which is attributed to the altered membrane composition resulting from elevated 18:1 content (Chen and Thelen 2013; Matos et al. 2007; Routaboul et al. 2012). In addition, because of differences in membrane physical properties, *fad2* mutants have been widely studied in the context of environmental stresses, such as heat and salinity, in addition to cold stress (Nguyen et al. 2019; Rodríguez‐Vargas et al. 2007; Xiao et al. 2022; Zhang et al. 2012).

From an applied perspective, FAD2 research has also been conducted on several crops. The identification and editing of *FAD2* genes in oilseed crops, such as sunflowers and peanuts (Blume et al. 2024; Dvorianinova et al. 2023; Neelakandan et al. 2022; Rozhon et al. 2023) have enabled the development of seed oils with improved storage stability and suitability for human consumption. Studies on *GmFAD2‐1A* and *GmFAD2‐1B* have been particularly well established (Pham et al. 2010). Although soybean is a representative oilseed crop capable of producing large amounts of oil, its native fatty acid composition is not optimal for consumption or storage (Lichtenstein et al. 2006; Oliva et al. 2006). To address this limitation, *FAD2* editing in soybeans has been used to reduce naturally high PUFA levels and increase 18:1 composition, thereby enhancing its industrial and commercial value.

However, the effects of multiplex CRISPR editing across a broader set of duplicated *GmFAD2* and *GmFATB* genes have not been systematically characterized using a field‐based allelic series.

In this study, we investigated these aspects by targeting a broader set of *GmFAD2* in combination with *GmFATB*. Although not intended, in‐frame deletion mutants were generated, and lines harbouring these mutations exhibited relatively lower 18:1 composition and somewhat higher levels of PUFA than the knockout mutants. In addition, the *2‐1a′b/‐/‐* and *2‐1ab′/‐/‐* lines, which carried in‐frame deletions in *GmFAD2‐1A* and *GmFAD2‐1B*, respectively, showed differences in fatty acid composition, with 18:1 composition of 77.7%, 72.7%, 18:2 composition of 4.9%, 4.7%, and 18:3 composition of 8.5%, 6.2%, respectively (Figure S2). These results suggest that GmFAD2‐1A and GmFAD2‐1B may contribute differently to 18:2 fatty acid synthesis.

Furthermore, comparison of the 18:1 composition among the *2‐1ab/‐/‐*, *2‐1ab/2‐2c/‐*, and *2‐1ab/2‐2bc/‐* lines (Figure 3A,B) showed values of 83.3%, 86.3%, and 87.0%, respectively, indicating that the proportion of 18:1 increased with the number of edited *GmFAD2* genes. However, when comparing the fatty acid compositions of *2‐1ab/2‐2c/‐* and *2‐1ab/2‐2ce/‐*, as well as between *2‐1ab/2‐2c/1a* and *2‐1ab/2‐2ce/1a* (Figure 3B), editing of *GmFAD2‐2E* did not appear to have any measurable effect on the seed fatty acid composition. In addition, because the public soybean genome data annotates only partial sequences of GmFAD2‐2E lacking a start codon, this gene may be nonfunctional.

Ultimately, the *2‐1ab/2‐2bc/1a* line, in which *GmFAD2* genes and *GmFATB1a* were simultaneously targeted, showed reduced SFA and PUFA contents, with an average 18:1 composition exceeding 90%. These results suggest that achieving ultra‐high oleic levels above 90% requires targeting seed‐specific *GmFAD2‐1* genes and co‐targeting oleic acid‐related genes such as *GmFAD2‐2B*, *C* and *GmFATB1a*.

In the case of *GmFATB*, only a single knockout of *GmFATB1a* was obtained in all Williams82‐background lines, and no fixed lines with simultaneous editing of *GmFATB1a* and *GmFATB1b* were advanced to subsequent generations. Double‐knockout individuals of *GmFATB1a* and *GmFATB1b* exhibited a male sterility (MS) phenotype, consistent with the previously reported *fatb1a:fatb1b* phenotype (Ma et al. 2021) (Figure S7). However, in lines developed on the Maverick‐background, individuals in which both *GmFATB1a* and *GmFATB1b* were edited and fixed (*2‐1ab/‐/1ab* type) produced seeds (Table 1). The seed fatty acid profile of this type showed a low total SFA content of 7.8% (16:0 and 18:0 at 4.9% and 2.9%, respectively), representing a more than 50% reduction compared to the wild‐type Maverick. In addition, the 18:1 composition in *2‐1ab/‐/1ab* line reached 89.1%, the highest level achieved among *GmFAD2‐2* targeted lines and *GmFATB* targeted lines (Figure S2B,C). These results provided further evidence for the contribution of *GmFATB1a* and *GmFATB1b* to the enhancement of 18:1 composition. At the same time, the absence of the MS phenotype in Maverick‐background double‐edited lines suggests the need for further investigation.

Qualitative seed fatty acid analysis revealed a reduction in SFA in lines where only *GmFAD2* was knocked out, a trend that has been consistently reported in other FAD2 studies (Bai et al. 2019; Buhr et al. 2002; Graef et al. 2009; Kinney 1996; Lee et al. 2021; Park et al. 2021; Shi et al. 2025; Tian et al. 2020). Additional quantitative GC analysis was performed to further characterize this phenomenon and confirm that SFA accumulation was reduced. Among SFA, 16:0 fatty acid was more strongly affected than 18:0 fatty acid (Figure 4C). A previous study proposed that the enlarged 18:1‐CoA pool may reduce 16:0‐CoA incorporation into TAG through competition at acyltransferase steps (Kinney 1996). However, in this study, gene expression analysis showed that *GmFATB* expression was reduced in *GmFAD2*‐exclusive knockout mutants compared to that in the WT (Figure 4D). These findings suggest that reduced *GmFATB* expression may contribute to the decreased SFA accumulation observed in *GmFAD2*‐edited lines, in addition to the previously proposed acyl‐CoA competition mechanism. However, the relative contribution of these mechanisms and the regulatory link between *GmFAD2* disruption and *GmFATB* expression require further investigation.

Correlation analysis of 805 GC datasets from *T*_4_ to *T*_5_ Williams82‐background lines characterized the relationship between 18:1 composition and seed TFA content (Figure 5; Figure S3). Background‐ and genotype‐based comparisons further showed that Williams82‐derived high‐oleic lines had higher TFA content than the corresponding WT. Several studies have reported varied seed oil content in high‐oleic soybean. In high‐oleic, low‐saturate soybean lines derived from event 335‐13, seed oil content remained nearly unchanged across eight Nebraska environments compared with the parental line (Graef et al. 2009). In contrast, oil content in *FAD2‐1A/FAD2‐1B* high‐oleic lines varied by genetic background, increasing in 17D‐derived populations but decreasing in M23‐derived populations (La et al. 2014). In this study, Williams82‐background lines showed increased seed TFA content while Maverick‐background lines showed inconsistent seed TFA content across generations (Figure S4). These results suggest that the effect of the high‐oleic trait on seed TFA content is influenced by genetic background. However, differences in the type and extent of *FAD2* modification and environmental conditions may also contribute to the contrasting phenotypes, although their individual contributions remain to be determined.

Given the consistently higher TFA content observed in the Williams82‐background lines, gene expression analysis was conducted to investigate potential metabolic factors associated with this phenotype, focusing on two pathways: TAG assembly and lipid turnover during late seed maturation (Figure 6). In the TAG assembly pathway, no consistent increase was observed in the expression of *GmDGAT1A/B/C*, *GmDGAT2D*, and *GmPDATA/B*. In contrast, *GmSDP1*, which is involved in lipid turnover, showed reduced expression in all lines except *GmSDP1‐2*, which showed very low expression, and *GmSDP1‐4* in the *2‐1ab/‐/1b* line.

Recent studies reported that GmSDP1 is involved in gluconeogenic activity during the late stages of soybean seed development (Dastmalchi 2021; Kambhampati et al. 2021). During this process, 18:1 fatty acid is preferentially degraded from TAG and directed into the β‐oxidation pathway. Accordingly, RNAi‐mediated knockdown of *GmSDP1* increases the TAG content in soybean seeds compared to that in the WT (Kanai et al. 2019). In addition, studies in *A. thaliana* have shown that *AtSDP1* expression is repressed by AT‐hook motif‐containing nuclear‐localized protein 5 (*AtAHL4*) and that PA can bind to AtAHL4 to relieve this repression (Cai et al. 2020). However, a regulatory mechanism involving AtAHL4 was associated with seedling establishment. Recent studies reported that during seed development, AtPHL7, rather than AtAHL4, is involved in *SDP1* regulation. In contrast to AtAHL4, AtPHL7 promotes *AtSDP1* expression; however, when bound to PA, it suppresses *AtSDP1* expression (Tang et al. 2026). These findings suggest that the reduced expression of *GmSDP1* observed in high‐oleic lines may be associated with PA‐dependent regulation involving AtPHL7 homologues in *Glycine max* .

To examine potential associations between reduced *GmSDP1* expression, PA metabolism, and AtPHL7‐homologous genes, the expression of four *GmPHR* genes (*GmPHR7*, *GmPHR14*, *GmPHR24*, and *GmPHR29*) was analysed (Figure S8). No consistent changes in *GmPHR* expression were observed, suggesting that the reduction in *GmSDP1* expression was not associated with transcriptional changes in these AtPHL7 homologues. Subsequently, genes involved in PA metabolism were analysed to determine whether PA‐related transcriptional changes accompanied the reduced *GmSDP1* expression. Although not all genes in the de novo PA synthesis pathway exhibited consistent changes, *GmLPAT14* expression was increased. In the lipid editing PA synthesis pathway, *GmPLDφ2* and *GmPLDα3* were upregulated. In addition, *DGK* genes (*GmDGK8*, *GmDGK9*, *GmDGK10*, *GmDGK11*, and *GmDGK12*), which have been reported to be associated with cold‐inducible PA accumulation in *Arabidopsis* (Arisz et al. 2013; Kue Foka et al. 2020), also showed increased expression compared to the WT (Figure 6C–E; Figure S6B). These results suggest that the reduction in *GmSDP1* expression may be associated with post‐transcriptional or translational regulation, potentially involving altered PA metabolism, rather than with transcriptional changes in AtPHL7‐homologous *GmPHR* genes. However, direct measurement of PA levels will be required to determine whether altered PA metabolism is involved in the regulation of *GmSDP1* expression.

PA accumulation in plants is known to be part of the cold stress response (Testerink and Munnik 2005; Yao and Xue 2018), and *fad2* mutants have been reported to be cold‐sensitive, exhibiting cold stress responses even under cool temperatures (Chen and Thelen 2013; Dar et al. 2017; Kargiotidou et al. 2008). Notably, during the cultivation of the *T*_4_ and *T*_5_ generations in 2024 and 2025, the late seed development stages (R7–R8), spanning from late September to mid‐October, experienced temperatures ranging from 20°C to 23°C (maximum) to 4.7°C–6.9°C (minimum) for approximately two weeks (Figure S9). Under the controlled 25°C (−R6.5) and 15°C (R7–R8) treatment, the high‐oleic line (*2‐1ab*/*2‐2c*/*1a*) showed higher TFA content than the WT on both a seed‐mass and per‐seed basis, reproducing the higher TFA phenotype observed in the field (Figure S10).

**FIGURE 7:**
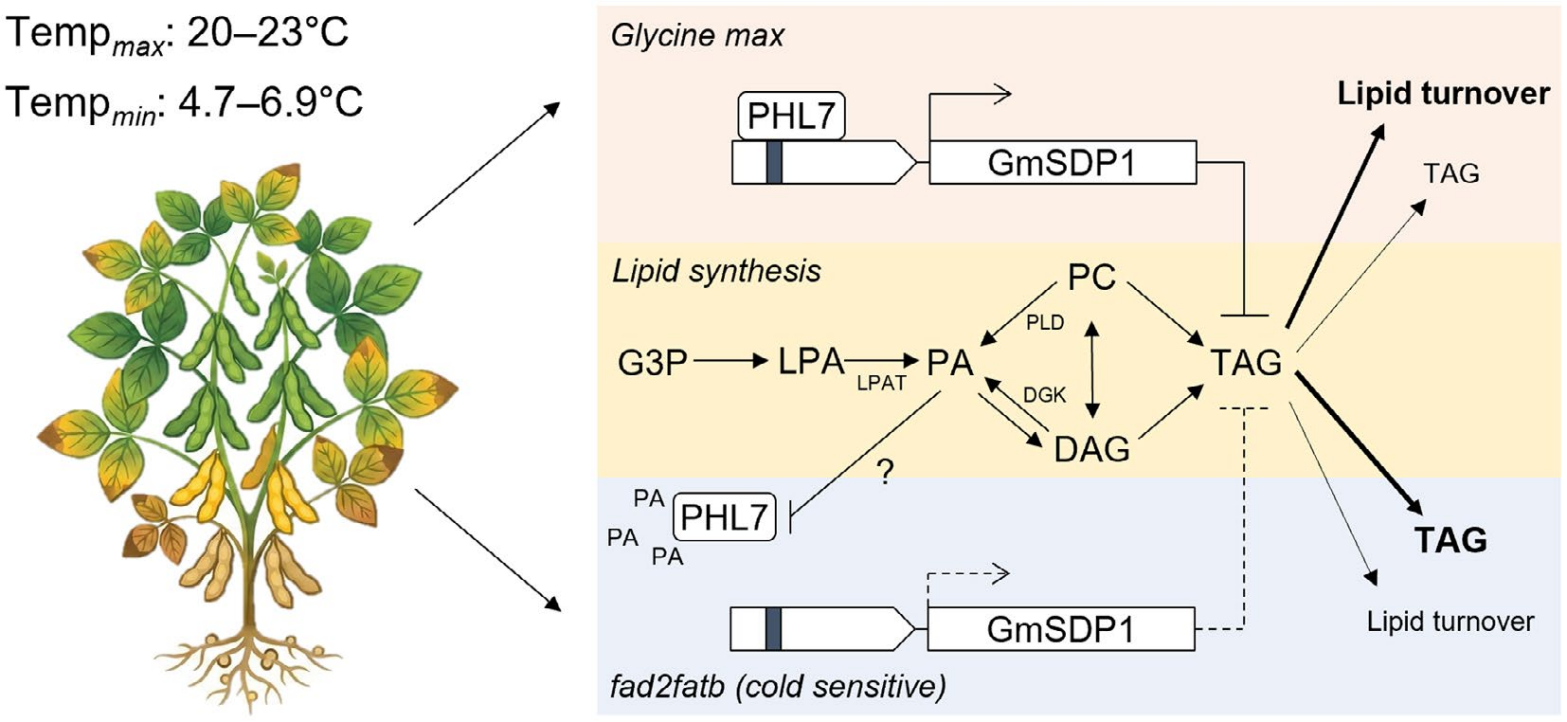
Proposed hypothesis for enhanced TFA accumulation in high‐oleic soybean lines. Mutations in *GmFAD2* and *GmFATB* alter seed fatty acid metabolism and increase oleic acid content. In Williams82‐derived high‐oleic lines, higher total fatty acid (TFA) content was accompanied by reduced *GmSDP1* expression and altered expression of several genes associated with phosphatidic acid (PA) metabolism. Field temperature records and the controlled cool temperature experiment further showed that the higher TFA phenotype was maintained under cool late‐developmental conditions. Based on these observations and previous reports of PA dependent regulation of *SDP1*, altered PA metabolism is proposed to be associated with reduced *GmSDP1* expression and lipid turnover, thereby potentially contributing to the higher TFA phenotype.

Taken together, the field temperature records, controlled temperature experiment, reduced *GmSDP1* expression, altered expression of PA metabolism related genes, and previous studies on PA accumulation and cold sensitive *fad2* mutant support a working hypothesis that cool conditions during late maturation stage may be associated with altered lipid turnover in high‐oleic soybean. Reduced *GmSDP1* expression may contribute to the higher TFA phenotype; however, direct measurements of PA levels and lipid turnover under controlled temperature conditions will be required to test this model (Figure 7).

In conclusion, this study identified the key gene targets required to achieve high 18:1 composition exceeding 90% through mutations of *GmFAD2‐1A/B*, *GmFAD2‐2B/C*, and *GmFATB1a*. Furthermore, the higher TFA content observed in high‐oleic lines was associated with reduced *GmSDP1* expression, suggesting a potential involvement of altered lipid turnover. These findings suggest that, beyond altering fatty acid composition, *FAD2* mutations may also be associated with changes in fatty acid accumulation and lipid turnover related gene expression during late seed development.

## 4 Materials and Methods

### 4.1 Plant Materials and Growth Conditions

Williams82 and Maverick were used as transformation backgrounds, with the corresponding wild‐type cultivars used as controls. Both the control and transgenic lines were grown in the field and in a greenhouse. For greenhouse growth, a 30 × 25 cm round pot was used. The soybean seeds were directly planted in soil 2–3 cm deep, watered every 2 days until the cotyledon appeared, grown for approximately 24 weeks, and harvested. The greenhouse temperature was maintained at 25°C. An LMO field located in KRIBB, South Korea, was used for transgenic soybeans from 2022 to 2025. The soybean seeds were directly planted in soil 2–3 cm deep with a 60 cm gap between adjacent plants. Sowing was performed in mid‐June, and harvesting was performed in early November. Temperatures from planting to harvest in 2024 and 2025 are shown in the supplementary data (Figure S9; Tables S13 and S14). For the controlled temperature experiment, wild‐type Williams82 and the high‐oleic line *2‐1ab*/*2‐2c*/*1a* were grown in a growth chamber under a 14‐h light/10‐h dark photoperiod. Plants were maintained at 25°C until the R6.5 stage and subsequently exposed to 15°C from R7 to R8 stage. Seeds were collected at the indicated developmental stages for fatty acid analysis.

### 4.2 Vector Construction and Soybean Transformation

pHEE401E_4sg, pBAtC_4sg, and pBAtC_6sg vectors were constructed using a modified Golden Gate assembly (Kim et al. 2021). The vectors were transformed into *E. coli* , spread on selection agar plates, and incubated for 16 h until a colony appeared. Colony PCR was performed to validate transformants using vector sequence‐based primers. Identified vector‐including colonies were inoculated into 4 mL of LB with selection antibiotics and incubated at 37°C for 16 h. Plasmid DNA was extracted from the cultures, and the freeze–thaw method was used to transfer the extracted vectors into *Agrobacterium tumefaciens* (GV3101), which were then spread on selection agar plates to select transformant colonies.

Transgenic soybean plants were generated using the half‐seed method (Kim et al. 2018) with seeds from the global cultivars Williams82 and Maverick. For Williams82, pHEE401E_4sg, pBAtC_4sg, and pBAtC_6sg vectors were used. For Maverick, only pHEE401E_4sg was used for transformation. Transformation experiments were conducted using three different vectors, each containing 130–150 soybean seeds. To screen for putative transformants expressing the Bar gene, the BASTA herbicide mixture (100 mg L^−1^ DL‐phosphinothricin (PPT) and Tween‐20) was applied to the upper surface of two trifoliate leaves of the transgenic (*T*_0_) plants. Herbicide response was assessed 3–5 days after PPT application. Plants exhibiting herbicide resistance were identified as putative transformants. The selected plants were grown continuously in a greenhouse, and the seeds were harvested for further analysis.

### 4.3 DNA Extraction and Next‐Generation Sequencing (NGS) Analysis

Genomic DNA of transgenic soybeans was extracted from young trifoliate leaf samples using the cetrimonium bromide (CTAB) method (Stefanova et al. 2013). Then genomic DNAs were diluted to 5 ng/μL and used as a template for nested PCR to generate an NGS library. Three rounds of nested PCR were performed to amplify the target genes and indices for the Illumina MiniSeq platform. The first round of nested PCR was used to narrow down the target genomic DNA sequence, thereby reducing redundant background. The second and third PCRs were performed to specify the Cas9 target sites for *GmFAD2* and *GmFATB* and a barcode sequence was added to the PCR product to classify the NGS results. The sets of first‐ and second‐round primers used for the nested PCR are listed in Table S15. The third PCR primer was the barcode index primer, which is generally used on the Illumina NGS platform. All target genes were indexed to individual plants. Each target gene from the same index was separated and identified using a target gene‐specific sequence. The indel ratios and mutant sequences were analysed using fastq‐join (Aronesty 2013) and CRIS.py (Connelly and Pruett‐Miller 2019).

### 4.4 RNA Extraction and RNA Expression Analysis

Total RNA was isolated from each sample using the LiCl RNA extraction method, and cDNA was synthesized using the TAKARA cDNA synthesis kit according to the manufacturer's protocol. From Williams82 (WT), leaves, R5 developing seeds (beginning seed, 45 DAF), and siliques were sampled and used for reverse transcription PCR (RT‐PCR); amplification was performed using Nex e‐Taq polymerase. The soybean *ACTIN* gene was used as a reference gene to compare band intensities. For the gene expression analysis, R7 developing seeds (beginning of maturity, 70 DAF) of *fad2fatb* mutants and Williams82 (WT) were sampled from three individual plants (*n* = 3). *Cons6* and *Cons7* (Libault et al. 2008) were used as reference genes, and primers for the target genes were designed using NCBI Primer‐BLAST. RT‐qPCR was performed with Takara TB green Premix on a StepOnePlus system (Applied bio) with three technical replicates, following the manufacturer's instructions. All the primer sequences are listed in Table S16.

### 4.5 Fatty Acid Analysis

The fatty acid composition and content of transgenic soybean seeds were analysed using GC. Cotyledon fragments from the developing or dry soybean seeds were sampled and weighed for fatty acid methyl ester (FAME) extraction. Each sample was mixed with 500 μL toluene and 1 mL of 5% H_2_SO_4_, containing pentadecanoic acid (15:0) as the internal standard and incubated for 2 h at 85°C in a water bath. Next, 2 mL of 0.9% NaCl and 250 μL of hexane were added. The mixture was centrifuged at 330 *g* for 2 min to separate the upper and lower phases. Subsequently, 200 μL of the upper hexane phase containing FAMEs was transferred directly to a GC vial for analysis (GC‐2030, Shimadzu, Kyoto, Japan) using a DB‐23 column (30 m × 0.25 mm, 0.25 μm film; Agilent, Santa Clara, CA, USA). The oven temperature increased by 5°C per minute from 190°C to 230°C and the analysis continued for 8 min until the 18:3 fatty acid peak was detected. Mole percentages (Mol%) were calculated based on the molecular weight of each fatty acid. Quantitative fatty acid content was indicated as fatty acid content per seed unit weight (μg mg^−1^ seed) or per seed (mg seed^−1^).

### 4.6 Correlation Coefficient Analysis

Through the *T*_4_ and *T*_5_ generations, 805 GC datasets obtained from wild‐type plants and each type of *fad2fatb* mutant with Williams82‐background were compiled for correlation coefficient analysis. Only quantitative variables were retained, and datasets containing missing values were excluded prior to statistical analysis. Pairwise correlations were calculated using Pearson's correlation coefficient (*r*) with the corresponding two‐sided *p*‐values to assess statistical significance. All correlation calculations were performed on the filtered dataset to maintain consistency across the comparisons. Data processing, statistical analyses, and visualization were conducted using AI‐assisted Python code with the Pandas NumPy, SciPy, Matplotlib, and StatsModels libraries. The code is presented in Supporting Information S1, and the data sets are presented in Tables S17 and S18.

## Author Contributions

Won Nyeong Kim and Hyun Uk Kim designed and supervised this study. Won Nyeong Kim, Yu‐Ri Choi, and Hye Jeong Kim performed the experiments. Won Nyeong Kim and Hyun Uk Kim analysed the data and performed the visualization. Won Nyeong Kim, Young‐Soo Chung, and Hyun Uk Kim wrote the manuscript. Won Nyeong Kim and Hyun Uk Kim revised the manuscript. All the authors contributed to the manuscript and approved the submitted version.

## Funding

This work was supported by grants from the National Research Foundation of Korea (NRF) (Grants RS‐2024‐00410854, RS‐2025‐00514459, and RS‐2025‐16069020), funded by the Ministry of Science and ICT (MSIT); the New Breeding Technologies Development Program (Grant RS‐2024‐00322277) of the Rural Development Administration (RDA); and the Korea Institute of Planning and Evaluation for Technology in Food, Agriculture and Forestry (IPET) (Grant RS‐2026‐25536374), funded by the Ministry of Agriculture, Food and Rural Affairs (MAFRA).

## Conflicts of Interest

The authors declare no conflicts of interest.

## Supporting information


**Supporting Information: S1.** Correlation coefficient matrix plot script.

**Figure S1:** Schematic progress of FAD2 and FATB gene‐edited transgenic lines from *T*_0_ to *T*_5_ generation.
**Figure S2:** Seed fatty acid profile of Williams82 wild‐type (WT) and *GmFAD2*/*GmFATB* mutants.
**Figure S3:** The correlation matrix of fatty acid profile in high‐oleic lines.
**Figure S4:** Seed TFA content of wild‐type (WT) and *GmFAD2/GmFATB* mutants.
**Figure S5:** Plant phenotypes of high‐oleic lines used for gene expression analysis.
**Figure S6:** Relative transcript levels of genes associated with lipid metabolism by RT‐qPCR in high‐oleic lines.
**Figure S7:** Segregating populations of *GmFATB1a* and *GmFATB1b* double knockout soybean lines and MS phenotype with predicted amino acid sequence.
**Figure S8:** Phylogenetic analysis of AtPHL7 and RT‐qPCR AtPHL7 homologous soybean genes in high‐oleic lines.
**Figure S9:** Temperatures of 2024 and 2025 and a schematic of the developmental stages of soybean.
**Figure S10:** TFA content in developing seeds of WT and high‐oleic lines under controlled cool temperature treatment.

**Table S1:** Indel frequencies in *T*_0_ transgenic soybean plants.
**Table S2:** Seed fatty acid composition of *T*_1_ progeny derived from *T*_0_ soybean plants.
**Table S3:** Indel frequencies in *T*_1_ transgenic soybean plants.
**Table S4:** Fatty acid composition of *T*_2_ seed.
**Table S5:** Indel frequencies in *T*_2_ transgenic soybean plants.
**Table S6:** Seed fatty acid composition of *T*_3_ progeny derived from *T*_2_ soybean plants (WT, Williams82; E4, pHEE401E_4sg; C4, pBAtC_4sg; C6, pBAtC_6sg).
**Table S7:** Indel frequencies in *T*_3_ transgenic soybean plants.
**Table S8:** Seed fatty acid composition and amount of *T*_4_ progeny derived from *T*_3_ soybean plants (WT, Williams82; pE4, pHEE401E_4sg; pC4, pBAtC_4sg; pC6, pBAtC_6sg; M, Maverick editing line).
**Table S9:** Indel frequencies in *T*_4_ transgenic soybean plants (M, Maverick editing line).
**Table S10:** Seed fatty acid composition and amount of *T*_5_ progeny derived from *T*_4_ soybean plants (WT, Williams82).
**Table S11:** Indel frequencies in *T*_5_ transgenic soybean plants.
**Table S12:** Indel sequence of edited target genes.
**Table S13:** Temperature of LMO field in 2024, KRIBB, Korea.
**Table S14:** Temperature of LMO field in 2025, KRIBB, Korea.
**Table S15:** List of oligonucleotide primers used for deep sequencing analysis.
**Table S16:** List of primers for RT‐qPCR.
**Table S17:** Correlation coefficient analysis GC data set 1.
**Table S18:** Correlation coefficient analysis GC data set 2.

## Data Availability

All data supporting the findings of this study are available in the paper and Supporting Information published online.
