# Supplementary material for: Multiplex FAD2/FATB Editing Generates Ultra‐High‐Oleic, Low‐Saturate Soybean With Increased Seed Fatty Acid Content
Source: Plant Biotechnol J. 2026 Sep 14;24(11):5930–45. doi: 10.1111/pbi.70758 (PMC13575352; doi:10.1111/pbi.70758)
Supplement: Supplementary file 1 [file PBI-24-5930-s001.docx]

<Supplementary data 1. Correlation coefficient matrix plot script.py>

```
import os
import pandas as pd
import numpy as np
import matplotlib.pyplot as plt
from scipy import stats
from statsmodels.nonparametric.smoothers_lowess import lowess

############################################################################################################################
# Run code in directory which including target ".xlsx" file. Result plot will generate in same directory.                  #
# ".xlsx" file must include variaty names in first row. The names will present as title of data histogram in matrix plot.  #
# Edit ".xlsx" in line 18 with user's file name                                                                            #
# Edit ".png" in line 85 to generate plot name. ".tiff" also available.                                                   #
# This code has no copyright. Modify as you need.                                                                          #
# If you use this code in your research or publication, please cite the original work. Thank you                           #
############################################################################################################################

cwd = os.getcwd()
data_path = os.path.join(cwd, ".xlsx")
df = pd.read_excel(data_path)

# exclude samples (ignore this function)
name_col = df.columns[0]
exclude = []
df2 = df[~df[name_col].astype(str).isin(exclude)]

num_df = df2.select_dtypes(include=[np.number]).dropna()
labels = list(num_df.columns)
data = num_df.to_numpy()
n = data.shape[1]

def p_to_star(p):
    if p <= 0.0001:
        return "****"
    elif p <= 0.001:
        return "***"
    elif p <= 0.01:
        return "**"
    elif p <= 0.05:
        return "*"
    else:
        return ""

fig, axes = plt.subplots(n, n, figsize=(2*n, 2*n))

for i in range(n):
    for j in range(n):
        ax = axes[i, j]

        if i == j:
            ax.hist(data[:, i])
            ax.set_title(labels[i], fontsize=14)

        elif i > j:
            x = data[:, j]
            y = data[:, i]
            ax.scatter(x, y, s=3, c="black")

            r, p = stats.pearsonr(x, y)
            lo = lowess(y, x, frac=0.4, return_sorted=True)

            if p >= 0.05:
                color = "gray"
            else:
                color = "red" if r > 0 else "blue"

            ax.plot(lo[:, 0], lo[:, 1], color=color, zorder=5)

        else:
            r, p = stats.pearsonr(data[:, j], data[:, i])
            star = p_to_star(p)
            ax.text(
            0.5, 0.5,
            f"{r:.2f}{star}",
            transform=ax.transAxes,
            fontsize=14,
            ha="center",
            va="center"
            )
            ax.set_xticks([])
            ax.set_yticks([])

        ax.tick_params(labelsize=6)

plt.tight_layout()
plt.savefig(os.path.join(cwd, ".png"), dpi=300)
plt.close()
```
